# The Vinculin-ΔIn20/21 Mouse: Characteristics of a Constitutive, Actin-Binding Deficient Splice Variant of Vinculin

**DOI:** 10.1371/journal.pone.0011530

**Published:** 2010-07-14

**Authors:** Susanna Marg, Ulrike Winkler, Marcello Sestu, Mirko Himmel, Madeleine Schönherr, Janina Bär, Amrit Mann, Markus Moser, Claudia T. Mierke, Klemens Rottner, Manfred Blessing, Johannes Hirrlinger, Wolfgang H. Ziegler

**Affiliations:** 1 Faculty of Medicine, Interdisciplinary Centre for Clinical Research (IZKF) Leipzig, University of Leipzig, Leipzig, Germany; 2 Faculty of Medicine, Carl-Ludwig-Institute for Physiology, University of Leipzig, Leipzig, Germany; 3 Faculty of Veterinary Medicine, Centre for Biotechnology and Biomedicine, University of Leipzig, Leipzig, Germany; 4 Department of Molecular Medicine, Max-Planck-Institute of Biochemistry, Martinsried, Germany; 5 Centre for Medical Physics and Technology, Friedrich-Alexander-University of Erlangen-Nuremberg, Erlangen, Germany; 6 Cytoskeleton Dynamics Group, Helmholtz Centre for Infection Research, Braunschweig, Germany; 7 Department of Nephrology, Hannover Medical School, Hannover, Germany; Institute of Infectious Disease and Molecular Medicine, South Africa

## Abstract

**Background:**

The cytoskeletal adaptor protein vinculin plays a fundamental role in cell contact regulation and affects central aspects of cell motility, which are essential to both embryonal development and tissue homeostasis. Functional regulation of this evolutionarily conserved and ubiquitously expressed protein is dominated by a high-affinity, autoinhibitory head-to-tail interaction that spatially restricts ligand interactions to cell adhesion sites and, furthermore, limits the residency time of vinculin at these sites. To date, no mutants of the vinculin protein have been characterized in animal models.

**Methodology/Principal Findings:**

Here, we investigate vinculin-ΔEx20, a splice variant of the protein lacking the 68 amino acids encoded by exon 20 of the vinculin gene VCL. Vinculin-ΔEx20 was found to be expressed alongside with wild type protein in a knock-in mouse model with a deletion of introns 20 and 21 (VCL-ΔIn20/21 allele) and shows defective head-to-tail interaction. Homozygous VCL-ΔIn20/21 embryos die around embryonal day E12.5 showing cranial neural tube defects and exencephaly. In mouse embryonic fibroblasts and upon ectopic expression, vinculin-ΔEx20 reveals characteristics of constitutive head binding activity. Interestingly, the impact of vinculin-ΔEx20 on cell contact induction and stabilization, a hallmark of the vinculin head domain, is only moderate, thus allowing invasion and motility of cells in three-dimensional collagen matrices. Lacking both F-actin interaction sites of the tail, the vinculin-ΔEx20 variant unveils vinculin's dynamic binding to cell adhesions independent of a cytoskeletal association, and thus differs from head-to-tail binding deficient mutants such as vinculin-T12, in which activated F-actin binding locks the protein variant to cell contact sites.

**Conclusions/Significance:**

Vinculin-ΔEx20 is an active variant supporting adhesion site stabilization without an enhanced mechanical coupling. Its presence in a transgenic animal reveals the potential of splice variants in the vinculin gene to alter vinculin function in vivo. Correct control of vinculin is necessary for embryonic development.

## Introduction

The cytoskeletal adaptor protein vinculin emerged together with cell adhesion receptors during early metazoan evolution [1]. It is highly conserved and expressed in all tissues. There is no gene homologous to the vinculin gene VCL in the genome. VCL gives rise to one confirmed alternative splice variant, metavinculin, which is preferentially expressed in muscle tissue [2], [3]. The vinculin protein is found in integrin receptor-based cell contacts, where it contributes to force transduction from the actin cytoskeleton to the membrane-apposed cell adhesion complex [4]. The targeting of vinculin to cadherin-based cell-cell contacts is less well understood, and in this case an involvement in the mechanical connection of actin filaments has been questioned [5]. Apart from influencing the mechanical stability of the cytoskeletal connection, vinculin interacts with numerous (protein) ligands and appears to fulfil a number of different functions in cell contacts, affecting regulation of adhesion complex turnover as well as signal transduction [6], [7].

Genetic inactivation (knock-out) of VCL leads to paralysis and defects in muscle architecture in *Caenorhabditis elegans* and to embryonal lethality in mice [8], [9]. Heart and brain defects observed in E8-E10 mouse embryos suggest defective cell migration, and/or reduced mechanical stability or turnover of contact sites. Developmental heart defects are the most likely cause of embryonal lethality, although other contributory factors have not been ruled out [9]. Cells derived from vinculin null animals form adhesion sites and display increased motility on 2D substrates [9], [10]. Consistently, in studies modulating vinculin expression, cells with high vinculin levels display reduced cell motility accompanied by low tumourigenicity, while those with low vinculin have increased motility and high tumourigenicity [11], [12]. This simple concept, however, has been challenged by recent reports showing that vinculin-deficient cells are less able to generate the traction force needed for the invasion of 3D collagen gels (or tissues) [13], [14]. The significance of vinculin function in tissue organization and mechanical stability of the heart muscle was demonstrated in heterozygous animals with one vinculin null allele and through heart-specific vinculin knock-out, which leads to cardiomyopathies and/or ventricular tachycardia causing sudden death of young mice [15], [16]. The characterization of other essential function(s) of vinculin requires further animal models.

Studies performed on vinculin null cells and animals indicate that the protein modulates adhesion and migration of cells and may define a critical point of regulation in the process of self-assembly and disassembly of adhesion sites. In fact, the protein's structure and biochemical properties suggest the importance of simultaneous regulation by several interaction partners to restrict high affinity interactions to adhesion sites [17], [18]. The vinculin protein consists of five alpha-helix bundles (Vd1-Vd5). The globular head (Vd1-Vd4) and the tail domain (Vd5, Vt) are connected by a flexible proline-rich linker and can establish an intramolecular, auto-inhibitory interaction with an estimated K_d_ of 1nM [17]. In the auto-inhibited conformation, high-affinity interactions with head ligands such as talin, alpha-catenin, and alpha-actinin and tail ligands like F-actin and acidic phospholipids, phosphatidyl-serine or phosphatidyl-inositol-4,5-bisphosphate (PIP_2_) are blocked. It has been suggested that the combinatorial activity of several partners as found only in cell contacts, is required to unlock the head-to-tail interaction (HTI) [17], [19]. Furthermore, the interaction of HTI-deficient, constitutive vinculin mutants with talin has been proposed to stabilize talin activity and to suppress adhesion site turnover. Thus, the critical impact of vinculin's intramolecular regulation on the cellular control of adhesion sites appears to be a consequence of its capacity to sustain the activation of talin and subsequently of integrin [20].

In this work, we analyse a constitutively active vinculin variant, vinculin-ΔEx20, using the knock-in VCL-ΔIn20/21 mouse model. Apart from vinculin knock-outs, no other mouse model investigating vinculin function has been analysed to date. Alternative splicing of exon 20 removes 2.4 helices from the Vt five-helix bundle domain, deleting the actin interaction sites as well as essential parts of the head interaction sites and promotes constitutive ligand binding of the vinculin head. Homozygous VCL-ΔIn20/21 animals die between embryonal day E10.5 to E12.5 with embryos showing exencephaly and lack of midline fusion. Furthermore, they express vinculin-ΔEx20 in addition to reduced levels of wild type vinculin. The phenotype of cells expressing vinculin-ΔEx20 reflects vinculin interactions in adhesion sites independent of auto-inhibition and cytoskeletal coupling, and contributes to a better understanding of vinculin functional regulation in cells and tissues.

## Results

### Embryonal lethality of the VCL-ΔIn20/21 mouse is linked to impaired splicing

**Figure 1:**
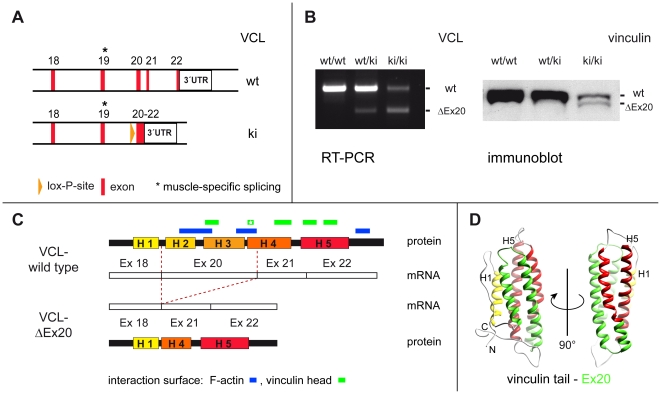
Impaired splicing leads to expression of vinculin-ΔEx20 in knock-in mice. (A) At the 3′end of the vinculin gene VCL, alterations were introduced for a knock-in approach. An endonuclease restriction site (BamH I) and a lox-P site are inserted into intron 19, 335 bp upstream of exon 20. The targeting construct continues with a short cDNA for exons 20, 21 and 22 (no introns) and the vinculin 3′UTR. (B) RT-PCR performed on total RNA isolated from E10.5 embryos with primers in exon 18 and 22 detects the expression of an additional shortened vinculin variant from the knock-in allele. Sequence analysis of the additional band reveals omission of exon 20. Splicing of vinculin exon 18 to exon 21 occurs in-frame and expression of the shortened protein variant, vinculin-ΔEx20, from the knock-in allele is detectable by vinculin-immunoblotting. (C) The vinculin tail consists of a bundle of five amphipathic α-helices (H1-H5) encoded by exons 18, 20, 21 and 22 of the VCL. Use of exon 19 is restricted to the muscle-specific splice variant metavinculin. Incorrect splicing skipping exon 20 (amino acids G916-Q983) leads to the deletion of vinculin tail helices H2 and H3 as well as a fragment of helix H4. These regions are involved in interactions of the tail with the vinculin head (green, [17], [18]) and F-actin (blue, [23]), as indicated. The position of mutations in vinculin-T12, a mutant with severely compromised head-to-tail interaction (HTI) [18], is marked (star). (D) Ribbon model views of the vinculin tail five-helix bundle showing the position of amino acids encoded by exon 20 highlighted in green [22].

To introduce selected mutations at the 3′ end of the vinculin gene, we generated a targeting vector for homologous recombination, eliminating introns 20 and 21 (Fig. 1A). The knock-in allele, referred to as VCL-ΔIn20/21, was designed with the intent to maintain normal expression of vinculin mRNA and protein, and subsequently, to allow Cre recombinase-controlled introduction of mutations in exons 20 to 22 at the C-terminus of vinculin (suppl. information, Fig. S1). Correct recombination was confirmed in targeted 129SV embryonic stem cells and a knock-in mouse line was established (suppl. information, Fig. S1). Unexpectedly, during breeding of these knock-in mice no homozygous (ki/ki) VCL-ΔIn20/21 animals were born. This indicated embryonal lethality of the VCL-ΔIn20/21 mouse. To analyze at which time point in development homozygous VCL-ΔIn20/21-animals die, embryos of different ages were isolated and genotyped. Indeed, homozygous embryos were detectable up to E12.5 but not at later stages (Table 1). To check whether lethality resulted from a lack of vinculin-mRNA transcription from the VCL-ΔIn20/21-allele, RT-PCR-analysis was performed on RNA isolated from E10.5 embryos of the different genotypes. This analysis using primers in exons 18 and 22 revealed the presence of the wild type mRNA as well as an additional alternatively spliced vinculin mRNA lacking exon 20 in both homozygous (ki/ki) and heterozygous (ki/wt) VCL-ΔIn20/21 embryos (Fig. 1B). The identity of this mRNA lacking exon 20 was confirmed by cloning and sequencing (suppl. information, Fig. S2). As exon 19 is alternatively spliced and used exclusively in the muscle-specific variant metavinculin, skipping of exon 20 effectively combines exons 18 and 21 without altering the reading frame. Analysis of exon 20 revealed the presence of consensus nucleotide sequences required for conventional intron splicing (suppl. information, Fig. S2). Thus, translation of vinculin-ΔEx20 mRNA results in a vinculin variant harbouring a defined deletion of 68 amino acids (encoded by exon 20) equivalent to 8 kDa. The extent of the deletion in vinculin tail is illustrated in Fig. 1C and D for the gene and the protein domain fold, respectively. Immunoblot analysis performed on single embryo protein extracts with a vinculin head specific antibody (hVin-1, Sigma) confirmed expression of a vinculin variant of reduced molecular weight in VCL-ΔIn20/21 (ki/ki) embryos. This protein was coined vinculin-ΔEx20 (Fig. 1B). Interestingly, we did not observe additional (proteolytic) protein bands. Hence in tissue extracts, there was no indication for enhanced proteolytic sensitivity of the vinculin-ΔEx20 variant. In mouse embryonic fibroblasts (MEFs) derived from VCL-ΔIn20/21 embryos, the half life of the ΔEx20 form is shorter than that of wild type vinculin and was estimated at 1.5 days (35 hours) (Fig. S3). Furthermore, total expression levels of wild type vinculin in homozygous embryos at embryonal day E10.5 were reduced as compared to heterozygous and to wild type littermates (Fig. 1B).

**Table 1:** Embryonic allele distribution in the VCL-ΔIn20/21 mouse line.

	wt/wt	wt/ki	ki/ki	n
**E 9.5**	16%	53%	31%	19
**E 10.5**	26%	51%	23%	78
**E 11.5**	32%	43%	25%	44
**E 12.5**	32%	48%	20%	25
**P 0**	38%	62%	0%	173

Genotype distribution was determined at different developmental stages. Homo­zygous VCL-ΔIn20/21 embryos were detectable until embryonal day E12.5. No homozygous animals were born.

### Phenotyping of homozygous VCL-ΔIn20/21 (ki/ki) embryos

**Figure 2:**
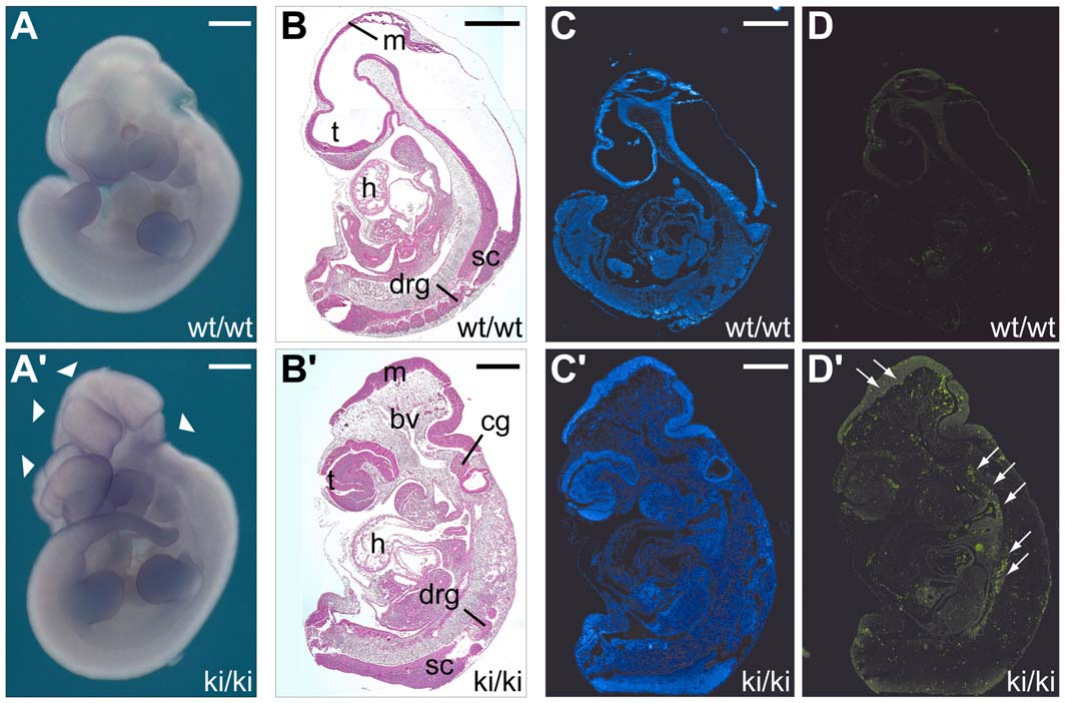
Homozygous VCL-ΔIn20/21 embryos are not viable. (A, A′) Lateral view of whole wild type and VCL-ΔIn20/21 embryos at E10.5. In the homozygous (ki/ki) VCL-ΔIn20/21 embryo (A′), exencephaly resulting from a neural tube closure defect is shown (arrow heads). (B, B′) Haematoxylin-Eosin staining of sagittal sections from wild type and VCL-ΔIn20/21 embryos at E10.5. m: mesencephalon; t: telencephalon; h: heart; sc: spinal cord; drg: dorsal root ganglia; cg: cranial ganglia; bv: blood vessels. Corresponding nuclear (C, C′) and TUNEL (D, D′) staining from wild type and VCL-ΔIn20/21 embryos at E10.5. Note increased cell death in VCL-ΔIn20/21 embryos (arrows). Scale bars: 500 µm.

As described above, VCL-ΔIn20/21 (ki/ki) embryos could be identified until E12.5 but not at later stages. Furthermore, E12.5 embryos were usually already dead and showed early signs of resorption. At E10.5 VCL-ΔIn20/21 (ki/ki) embryos were still alive and showed the same size as wild type or heterozygous embryos isolated from the same pregnant mouse, indicating that embryonic death occurred between E10.5 to E12.5. Morphological inspection of VCL-ΔIn20/21 (ki/ki) embryos revealed a severe craniofacial defect. The cranial neural folds failed to fuse resulting in the formation of an exencephaly. Furthermore, other head structures also failed to fuse at the ventral cranial midline (Fig. 2A′). Histological investigation of E10.5 embryos confirmed the absence of ventricles, a phenotype that has also been described for vinculin knock-out embryos. In addition, the head mesenchyme was loosely organized and contained massively dilated blood vessels (Fig. 2B′). However, histological analysis of all other inner organs such as heart, liver, spinal cord and gut as well as limb buds, dorsal root and cranial ganglia showed neither a developmental delay nor any obvious defect (Fig. 2 B′ and data not shown).

To test whether expression of the vinculin-ΔEx20 variant affects cell survival during development, TUNEL-staining on histological sections from E10.5 embryos was performed. Indeed, whereas only a few TUNEL positive cells were present in sections from wild type embryos, a massive increase in TUNEL positive cells could be detected in VCL-ΔIn20/21 (ki/ki) embryos. Interestingly, massive cell death was found almost exclusively in mesenchymal tissue, ventral to the spinal cord and in the neuroepithelium, but was not observed in heart or liver (Fig. 2 D′).

### Effects of vinculin-ΔEx20 on cell adhesion sites

**Figure 3:**
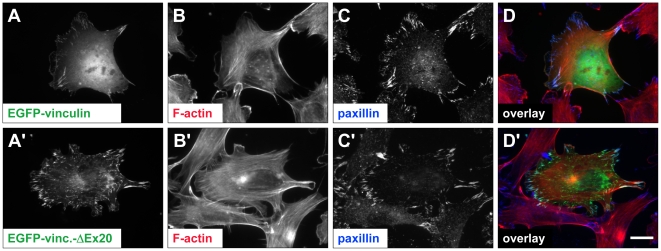
Vinculin-ΔEx20 expression affects morphology and distribution of adhesion sites. Localization of EGFP-vinculin wild type (A–D) and EGFP-vinculin-ΔEx20 (A′–D′) in C2C12 cells. Paxillin staining is employed as an independent marker for integrin-based adhesion sites (C, C′). In EGFP-vinculin-ΔEx20 expressing cells, the number and size of central adhesions appear to be increased. Note the reduction of diffuse cytoplasmic staining (A′). Scale bar: 20 µm.

To evaluate the potential effects of the vinculin splice variant on embryonal development, we generated a vinculin-ΔEx20 expression plasmid equipped with an N-terminal EGFP for expression and detection in eukaryotic cells. In C2C12 myoblasts, the efficiencies of transfection and expression levels of the wild type and ΔEx20 forms of GFP-tagged vinculin as determined by FACS analysis were identical (data not shown). Immunoblots revealed ratios of 1∶1.2–1.8 of endogenous to ectopically expressed protein and did not show any additional short fragments that would be indicative of enhanced degradation of the vinculin-ΔEx20 (data not shown). Analysis of protein targeting in transfected C2C12 cells revealed strong adhesion site localization concomitant with a reduced cytoplasmic occurrence of vinculin-ΔEx20 compared to vinculin wild type (Fig. 3). Furthermore, contact sites containing vinculin-ΔEx20 appeared to be larger, and more prevalent in the central regions of transfected cells.

**Figure 4:**
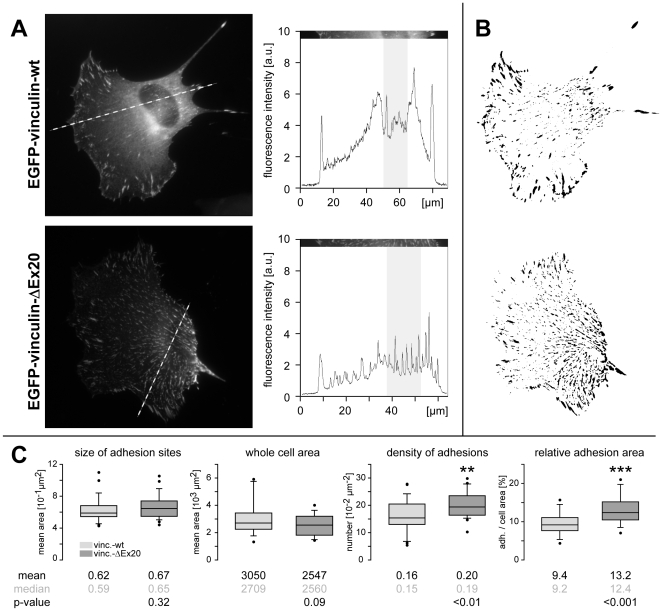
Vinculin-ΔEx20 effects on the size and density of adhesion sites. (A) Line scans (broken line) taken from fluorescence images provide intensity profiles (right), which allow comparison of fluorescence signals in adhesion sites and the cytoplasm. Note the strong increase in basal fluorescence intensity adjacent to the nucleus (shaded) of the vinculin wild type but not the vinculin-ΔEx20 expressing C2C12 cell. (B) Processing of fluorescence images (see material and methods) yields whole cell areas and binary footprints of adhesion sites as illustrated. (C) Footprints taken from C2C12 cells expressing vinculin wild type (n = 26) or vinculin-ΔEx20 (n = 28) are used to quantitatively assess number and size of adhesion sites in relation to the spreading area of cells. Box plots indicate median values and capture 50% of data in boxes and 80% in whiskers. Mean values, median and statistical significance (t-test) are given underneath. Note the highly significant increase in adhesion density and relative adhesion area of vinculin-ΔEx20 expressing C2C12 cell. p-value: **<0.01, ***<0.001.

These alterations of adhesion sites were analysed quantitatively by determining the average adhesion size and total adhesion areas in relation to whole cell areas of C2C12 cells, expressing low and comparable levels of EGFP-tagged wild type or ΔEx20 vinculin. In fluorescence images, line profiles confirmed the presence of a strong cytoplasmic pool of wild type vinculin leading to a pronounced perinuclear signal, which was absent in cells expressing vinculin-ΔEx20 (Fig. 4A). Adhesion site footprints were extracted from binary transformed fluorescence images (Fig. 4B). Analyses of footprints from cells expressing either vinculin wild type or vinculin-ΔEx20 (n≥26 cells and ≥11000 contacts in each case) reported a comparable size distribution of adhesion sites with average adhesion size of 0.62±0.03 µm^2^ (mean ± SEM) and 0.67±0.03 µm^2^, respectively (Fig. 4C). In vinculin-ΔEx20 expressing cells, the slight increase in adhesion size (7%) was accompanied by a reduced whole cell area (17%), decreasing from an average of 3.0±0.3×10^3^ to 2.5±0.2×10^3^ µm^2^ (mean ± SEM). Taken individually, neither characteristic showed statistically significant differences with p- values (t-test) of 0.32 and 0.09, respectively. However, analysis of adhesion relative to whole cell areas confirmed the initial (subjective) impression. The density of adhesion sites rose from 16±1 to 20±1 sites in 100 µm^2^ (mean ± SEM; p<0.01) and the adhesion area reached 13.2±0.7% of the total area of the cell (mean ± SEM; p<0.001), reflecting an increase of 40% in vinculin-ΔEx20 compared to vinculin wild type expressing cells (Fig. 4C). Hence, quantitative data confirmed significantly enhanced adhesion following vinculin-ΔEx20 expression. This is reminiscent of effects reported for expression of the vinculin head domain [20] and suggests constitutive binding of vinculin-ΔEx20 to talin and other ligands of the vinculin head.

### Consequences of vinculin-ΔEx20 expression in mouse embryonic fibroblasts

**Figure 5:**
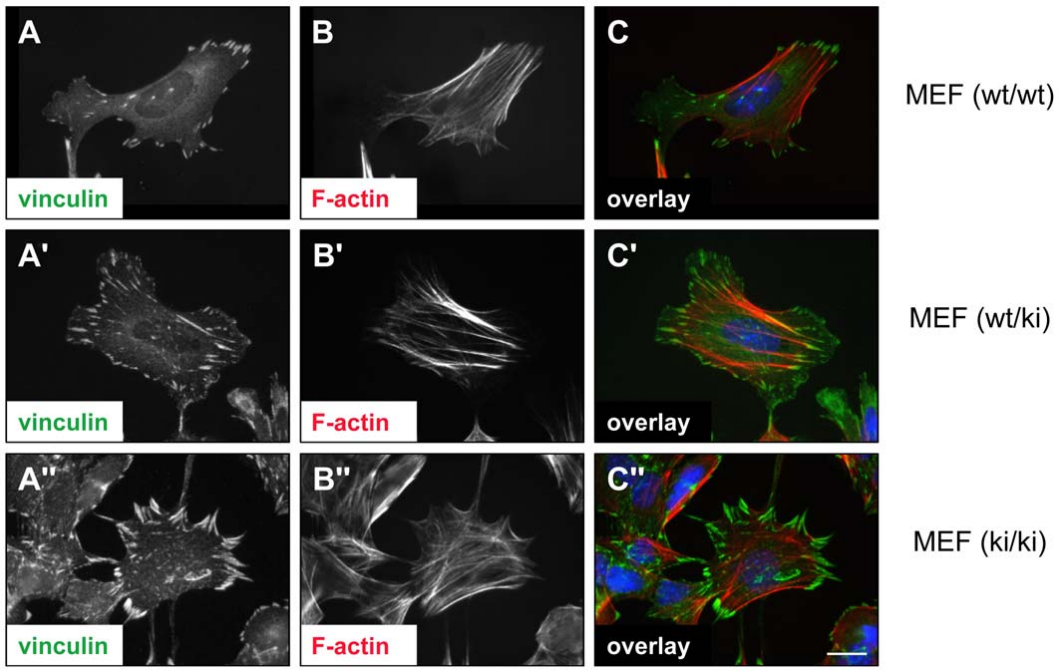
Distribution of endogenous vinculin in VCL-ΔIn20/21 MEFs. Vinculin localization (A-A″; immunostaining) and actin filaments (B-B″; phalloidin staining) are depicted in mouse embryonic fibroblasts (MEFs) derived from E10.5 embryos. Cells from VCL-ΔIn20/21 littermates of all genotypes, (wt/wt) (A–C), (wt/ki) (A′–C′), or (ki/ki) (A″–C″) are analyzed. In vinculin-ΔEx20 expressing MEFs, central adhesions appear pronounced and the cytoplasmic staining is reduced. Heterozygous (wt/ki) MEFs display an intermediate phenotype. Scale bar: 20 µm.

**Figure 6:**
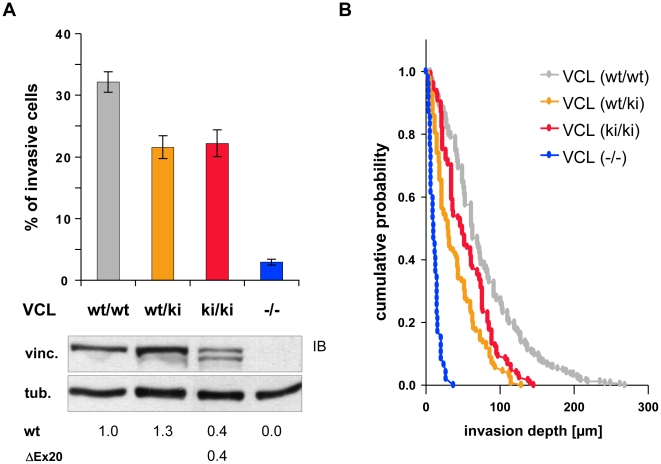
VCL-ΔIn20/21 (ki/ki) MEFs invade collagen gels. (A) Fibroblasts of different genotype were seeded onto three-dimensional collagen matrices (gels) and cultured for 3 days. In vinculin-deficient VCL (−/−) MEFs, the low percentage of invasive cells confirms strong inhibition of cell adhesion/invasion, as reported earlier [14], whereas transgenic MEFs, VCL-ΔIn20/21 (wt/ki) and (ki/ki), are reduced with respect to (wt/wt) controls. Variance analysis established significant differences between all genotypes except for (wt/ki) and (ki/ki) (post hoc Holm-Sidak, p<0.01). The immunoblot (IB) reveals vinculin protein levels, and numbers underneath indicate the average expression compared to (wt/wt) MEFs (n = 3). Note that vinculin expression levels of the different fibroblast genotypes do not show a linear correlation to invasiveness of cells. (B) Invasion profiles expressed as cumulative probability of finding a cell at or below a given invasion depth reveal that (wt/ki) and (ki/ki) MEFs invade with similar efficiency, and (wt/wt) MEFs invade deeper. Invasion of VCL (−/−) MEFs is almost entirely inhibited. Error bars: SEM.

To address the influence of vinculin-ΔEx20 on vinculin function in homozygous VCL-ΔIn20/21 embryos, primary MEFs from E10.5 embryos were cultured. In these cells, as in the transfection experiments, immunostaining of endogenously expressed vinculin revealed distinct localization i.e. low perinuclear intensity and enlarged adhesion sites in VCL-ΔIn20/21 (wt/ki) and (ki/ki) MEFs as compared to VCL (wt/wt) MEFs from littermates, confirming the impact of vinculin-ΔEx20 on the cellular control of adhesion site formation (Fig. 5). Comparable effects were observed upon expression of vinculin-ΔEx20 in vinculin knock-out MEFs and other cell lines (data not shown). To further investigate the extent of the dominant ΔEx20 effect, immortalized MEFs of different genotypes, wild type, VCL null (−/−), and VCL-ΔIn20/21 (wt/ki) and (ki/ki), were generated. The migratory behaviour of the different fibroblast populations was determined, using an invasion assay that addresses key functional aspects of cell motility in three-dimensional collagen matrices (collagen gels) [14]. In this assay, vinculin-deficient cells served as negative control as they generate low contraction forces [12] and cannot invade collagen gels mechanically, which is not a consequence of reduced matrix protease expression or activity [14]. As shown in Figure 6, the fraction of invasive vinculin null cells was very low with 2.9±0.5% (mean ± SEM), while properties of VCL-ΔIn20/21 transgenic cells, (wt/ki) and (ki/ki), were similar with 21.6±1.8% and 22.2±2.2% of invading cells, respectively. Remarkably, the significant reduction in invasive cells (p<0.01) that was observed with respect to vinculin (wt/wt) MEFs (32.1±1.7%) did not correspond to expression levels of vinculin protein (Fig. 6A). Heterozygous (wt/ki) MEFs expressed more vinculin than wild type MEFs, whereas (ki/ki) cells expressed 40% of wild type and a similar amount of ΔEx20 vinculin. Unlike the embryos (see Fig. 1B), MEFs in cell culture adjusted their vinculin expression, and it appears that for cellular invasiveness there is an optimal expression level of vinculin protein. Moreover, invasion profiles, which reflect the average depth of invasion (Figure 6B), confirmed the invasion characteristics of the different genotypes. The average depth of invasion was 76.0±2.2 µm (mean ± SEM) for (wt/wt) MEFs, 39.8±2.9 µm and 54.9±3.1 µm for transgenic (wt/ki) and (ki/ki) MEFs, respectively, and 11.9±1.0 µm for vinculin null (−/−) MEFs. In contrast to other constitutive mutants like vinculin-LD that block migration at low expression rates [21], there is no indication for a dominant negative effect of vinculin-ΔEx20 expression on cell migration in the three-dimensional matrix environment.

### Ligand binding of vinculin-ΔEx20

**Figure 7:**
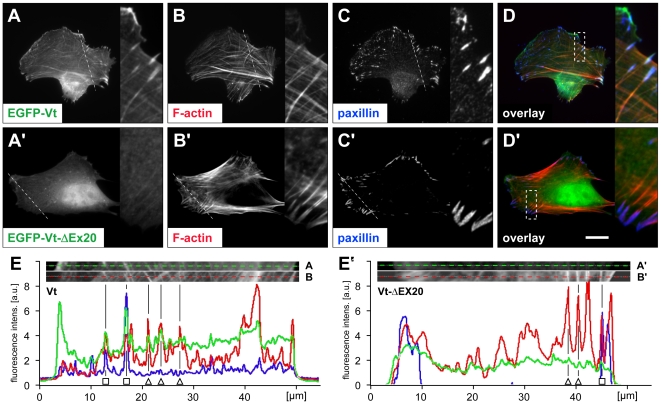
Actin filament binding of vinculin tail (Vt)- ΔEx20 is defective. Co-localization studies of EGFP-tagged vinculin tail (aa 879–1066) and F-actin in C2C12 cells show stress fibre decoration and adhesion site localization for the wild type tail (A–D) and a predominantly diffuse distribution of vinculin tail-ΔEx20 (A′–D′) confirming differences in actin filament interaction. Enlargements (2,3x) refer to boxed areas in (D) and (D′), respectively. Paxillin staining marks adhesion sites (C, C′). Scale bar: 20 µm (8.6 µm enlargement). (E, E′) Line profiles taken from all three protein channels (A–C/A′–C′; broken lines) reveal correlation of EGFP-Vt (E), but not of EGFP-Vt-ΔEx20 (E′), with F-actin and paxillin intensity distributions. Selected adhesion sites (open squares) and actin stress fibres (open triangles) are marked.

Having characterized vinculin-ΔEx20 as a constitutive variant that can enhance adhesion site occurrence, most likely as a consequence of a reduced head-to-tail interaction, we next addressed the impact of ligand binding to the vinculin-ΔEx20 tail domain. Humphries et al. suggested selective targeting of the vinculin tail (Vt) to a subset of actin filaments [20]. The molecular details of such interaction, however, have not been resolved. In C2C12 cells, expression of EGFP-tagged Vt (amino acids 879–1066) carrying the F-actin and lipid binding site(s) of the protein [22], [23], revealed adhesion site localization and some decoration of actin stress fibres by wild type vinculin tail (Fig. 7A–D). The positions of adhesion sites were marked by paxillin staining. When vinculin tail-ΔEx20 was expressed, EGFP-staining was much more diffuse, indicating reduced (lateral) binding to actin filaments (Fig. 7A′–D′). Line profiles of transfected cells confirmed the correlation of the EGFP-Vt wild type with actin filaments and paxillin signals, while no such correlation was evident for the EGFP-Vt-ΔEx20 signal, indicating disruption of targeting (Fig. 7E, E′).

**Figure 8:**
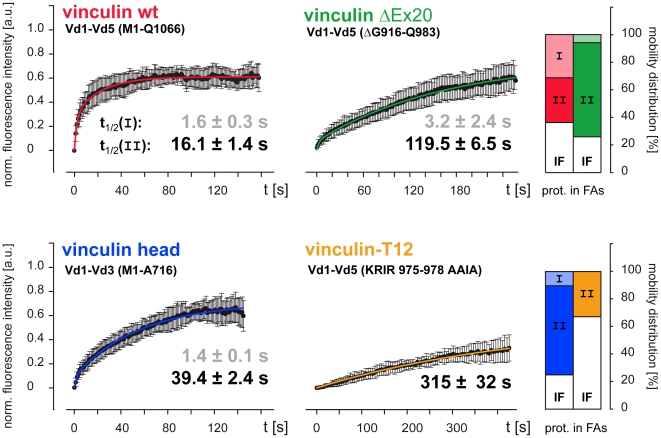
Residency times of constitutively active vinculin variants in focal adhesions. Fluorescence recovery after photobleaching (FRAP) experiments using EGFP-tagged vinculin constructs were used to determine half life times of bound protein t_1/2_(II) (means ± S.D.) and the percentage of protein in the immobile fraction (IF) [24]. (I, II and IF refer to fractions of tethered, bound and immobile protein in adhesions, respectively; see Results). Comparison of vinculin wild type (red) with a vinculin head construct, Vd1–Vd3 (blue), the vinculin-ΔEx20 variant (green), and the HTI mutant vinculin-T12 [18] (orange) reveals significantly increased half lives of the different constructs in adhesion sites. Residency times t_1/2_(II) of the (constitutive) head construct, vinculin-ΔEx20, and vinculin-T12 were 2, 7 and 20 fold as long, respectively. The T12-mutant showed no tethered fraction (I). Thus, mono-exponential regression was calculated (t_1/2_(II) only). Note different time scales used on the x-axes. Vinculin head constructs and vinculin-ΔEx20 show comparable immobile fractions (IF), whereas the recovery of vinculin-T12 is very low. Reduced amounts of tethered protein (I) in the mobility distribution plot, as compared to wild type, indicate constitutive ligand binding. Error bars: S. D.

A strong influence of the vinculin-ΔEx20 tail domain on the cellular function of vinculin became evident when we investigated residency times of different EGFP-tagged variants by fluorescence recovery after photobleaching (FRAP). C2C12 cells were used for the comparison of vinculin kinetics, since we have previously obtained highly reproducible values in these cells with a number of different proteins and domain constructs [24]. Bi-exponential fit of fluorescence recovery curves indicated two kinetic pools of protein in adhesion sites with t_1/2_(I) corresponding to tethered protein and t_1/2_(II) reflecting the pool of actively bound protein [24]. Figure 8 reveals that the half life time t_1/2_(II) of 16±1 s (mean ± S.D.) for wild type vinculin is relatively low in C2C12 cells as compared to other cells, but well within the range of values reported for vinculin in the literature [21], [25], [26]. Consistent with results obtained in NIH3T3 cells [20], vinculin head domain constructs showed 2–3 times prolonged residency times in C2C12 cells, with t_1/2_(II) of 39±2 s and 49±3 s for vinculin head Vd1-Vd3 (amino acids 1–716) and vinculin head Vd1-Vd4+ (amino acids 1–880, profile not shown), respectively. Thus, the half life time t_1/2_(II) of 120±7 s determined for vinculin-ΔEx20, which is 7 times longer than that of vinculin wild type, demonstrated strongly enhanced binding of this vinculin splice variant to adhesion sites. This behaviour differed from that of (pure) vinculin head interactions. Most notably, the vastly prolonged residency time was not accompanied by a decrease of vinculin-ΔEx20 protein in the mobile fraction (74%). In contrast, vinculin-T12, an established head-to-tail binding mutant with defective Vd4-Vt(Vd5) interaction yet intact F-actin binding [18], showed a sharp reduction in its mobile fraction to 33% and t_1/2_(II) of 315±32 s in C2C12 cells (Fig. 8). These differences in protein behaviour presumably reflect enhanced ligand interaction of the intact tail domain in vinculin-T12, compared to that of vinculin-ΔEx20, which lacks the 68 amino acids of exon 20.

## Discussion

Here we report biochemical and functional consequences of the genetic removal of introns 20 and 21 from the vinculin gene VCL. Homozygous (ki/ki) embryos carrying the VCL-ΔIn20/21 allele, die between E10.5 to E12.5 and display severe craniofacial defects. Vinculin expression analysis in E10.5 embryos reveals reduced levels of the endogenous wild type protein and the appearance of an alternatively spliced vinculin variant lacking exon 20. Vinculin-ΔEx20 is a (calculated) 109 kDa protein devoid of the actin interaction sites of the tail (Vd5) and of the inhibitory intramolecular head-to-tail interaction (HTI) (Vd4–Vd5). Reduced HTI of vinculin-ΔEx20 leads to strongly enhanced residency times of the protein and stabilization of adhesion sites, affecting cellular control of adhesion and motility.

### The VCL-ΔIn20/21 mouse and consequences of VCL targeting

The phenotype of the VCL-ΔIn20/21 mouse only partially resembles the phenotype of vinculin knock-out animals, although both mouse mutants die during midgestation after having completed gastrulation. Both mouse mutants exhibit a cranial neural tube closure defect resulting in an exencephaly. As suggested by Xu et al. [9], this phenotype might be due to a defect in the regulation of the actin cytoskeleton, which is crucial for bending of the neural folds toward the midline. The strongly dilated blood vessels in the head most likely result from a failure in brain formation, as the head mesenchyme has more space and is therefore only loosely associated and less able to counteract the blood pressure.

Despite these similarities, the phenotype of the vinculin null embryos is more severe than that of the VCL-ΔIn20/21 embryos. Vinculin null embryos are already smaller at E8.5 and only some survive until E10.5 [9]. In addition, in vinculin null embryos the cranial nerves failed to form and the dorsal root ganglia were much smaller. Beside the neuronal defects, vinculin knock-out embryos show a severe malformation of the heart, which never initiated contraction [9]. The heart defect prevents normal nutrient supply and removal of waste products in the fast growing embryo and, thus, is the most likely cause of early death and dramatic developmental retardation in the knock-out embryos.

In contrast, VCL-ΔIn20/21 embryos show no growth retardation and are the same size as wild type embryos at E10.5. Consistently, histological analysis of hearts from VCL-ΔIn20/21 embryos revealed normal differentiation into epicardial, myocardial and endocardial structures. In addition, there was no obvious defect in the formation of the peripheral nervous system in these embryos. Thus, reduced levels (far below 50%) of wild type vinculin are probably sufficient to sustain normal organ development. Despite this absence of apparent phenotype, expression of vinculin-ΔEx20 protein and reduced expression of wild type vinculin cause apoptotic cell death in the neural epithelium and in mesenchymal cells ventral to the spinal cord. Whether or not this is a consequence of defective cell adhesion and migration remains unclear.

Studies on mice carrying vinculin null (knock-out) alleles provide evidence that one functional vinculin allele is sufficient to allow regular embryonal development. Heterozygous mice are fertile and display no obvious pathology. Investigating cardiac function in adult heterozygous vinculin null mice, Ross and co-workers report stress-inducible cardiac complications [15]. In these animals, vinculin protein levels in the heart are decreased by about 60%. Consistent with morphological observations in knock-out embryos [9], these findings suggest that a critical threshold concentration of vinculin is required to preserve functional integrity of at least some tissues. In adult heterozygous VCL-ΔIn20/21 mice, analysis of different organs indicates significant reduction of vinculin levels to 40–60% in otherwise normal animals (S. Marg, W. H. Ziegler, unpublished data). Since the minimum levels of vinculin allowing normal embryonal development have not been established and would require a mouse model with tuneable vinculin expression, we cannot fully exclude that lethality of homozygous VCL-ΔIn20/21 embryos is due at least in part to insufficient vinculin levels.

### Characteristics of the vinculin-ΔEx20 protein

Vinculin-ΔEx20 is detected as one protein without additional head fragments in immunoblots of primary MEF cells derived from VCL-ΔIn20/21 (ki/ki) mice and upon ectopic expression in different cell lines. In cells, this protein displays preferential adhesion site targeting together with a low cytoplasmic pool and induces a rise in adhesion site density. These characteristics indicate constitutive binding of vinculin-ΔEx20 to head ligands such as talin and alpha-actinin. Direct comparison of the ΔEx20-splice variant to vinculin head constructs or the head-to-tail binding mutant vinculin-T12 reveals informative differences. Ballestrem and co-workers report that a range of vinculin head constructs as well as vinculin-T12 can induce a 3–4 fold increase in total adhesion area covering more than 20% of the cell's spreading area. This drastic rise appears to result from a 2-fold increase in average adhesion size and a 2–3 fold rise in the number of adhesions [20]. In comparison, vinculin-ΔEx20 causes only moderate changes in number and size of adhesions leading to 13% of relative adhesion area or a 1.4 fold increase with respect to vinculin wild type expression. Differences in residency times, which strongly depend on the regulation of vinculin head interactions [25], are even more striking. In C2C12 cells, half life times of head domain constructs, Vd1–Vd3 and Vd1–Vd4+, are similar to the values reported for NIH3T3 cells [20]. In contrast, the half life of vinculin-ΔEx20 was 2.5–3 times longer than that of these head constructs, and vinculin-T12, which was more difficult to investigate due to low recovery, revealed 6.5–8 fold longer residency times. In conclusion, vinculin-ΔEx20 displays strong head binding accompanied by a moderate induction of cell contacts and no dominant negative effect on cell motility/migration.

Some studies indicate that half life times of vinculin (and vinculin head) in adhesions are directly linked to those of talin and integrins [20], [25], suggesting that vinculin modulates talin activity [27] and consequently talin-induced integrin activation [20], [25], while other proteins of the cell adhesion complex such as paxillin remain unaffected. Hence we can assume that (i) the expression of vinculin-ΔEx20 will enhance talin and integrin activation, leading to enhanced number and stability of adhesion sites, and that (ii) this effect is responsible for the functional consequences of exon 20 removal. Deletion of this exon from the vinculin tail five-helix bundle (see Fig. 1C, D) removes helices H2, H3 and a fragment of H4 including the two characterized interaction sites for actin [23], the interaction sites with domains Vd4 and Vd1 (partial) of vinculin head [17], [18] and the basic ladder of helix H3. Thus, vinculin tail-ΔEx20 lacks the characterized interaction sites for actin filaments and, similar to vinculin-T12, shows a strongly reduced autoinhibitory head-to-tail interaction. These changes can account both for the constitutive talin binding of the head domain in vinculin-ΔEx20 and for the poor or absent F-actin interaction of the tail domain.

Consequences for the interaction of vinculin tail-ΔEx20 with acidic phospholipids are less obvious. This interaction is functionally important for vinculin's activity and residency time in adhesions and competes for actin filament binding [6], [28]. Consistently, in cells expressing vinculin-LD, a mutant lacking the interaction with acidic phospholipids, spreading and motility defects were observed, which were linked to suppression of lipid-mediated adhesion site dissolution [21]. Structural data and mutagenesis indicate that several parts of the tail are involved in lipid interactions, the basic ladder (H3), the basic collar and the hydrophobic hairpin at the C-terminus [21], [26], [29]. In vinculin-ΔEx20, the basic ladder and a part of the basic collar but not the C-terminus are missing, suggesting impaired interaction of vinculin-ΔEx20 with acidic phospholipids. However, a recent study using C-terminal tail peptides stresses hydrophobic aspects of the membrane interaction [30] and the biophysical characterization of vinculin mutants in cells highlights the impact of the C-terminal peptide on lipid anchoring and traction formation [31]. Therefore, the C-terminus of vinculin-ΔEx20 should support membrane association and sustain vinculin's influence on mechanical properties of the plasma membrane. Since residency times observed for vinculin-ΔEx20 in adhesions are long compared to vinculin head constructs, it seems likely that adhesion site dynamics and function of the ΔEx20-splice variant are controlled by a combination of vinculin head and vinculin tail-lipid interactions.

Finally, the altered lipid binding site based on the basic collar and hydrophobic hairpin of vinculin tail may still be subject to regulation by protein phosphorylation. In the ΔEx20-tail, Y1065 may be phosphorylated via the FAK-src signalling axis, which is associated with enhanced adhesion turnover [32], and this might down regulate electrostatic interactions of the basic collar with the negatively charged membrane. Furthermore, FAK-mediated strengthening of adhesion sites requires talin and negatively affects steady-state levels of vinculin incorporation [33]. Thus, vinculin-ΔEx20 is expected to uncouple the activation of talin and integrins from the mechanical connection to the cytoskeleton, while maintaining responsiveness of vinculin for intracellular signalling via acidic phospholipids and phosphorylation. This regulation would account for the moderate effects on the cellular control of adhesion sites and migration observed upon expression of vinculin-ΔEx20.

### Tissue functions of vinculin

Vinculin is a phylogenetically conserved single-copy protein which, apart from providing unique functions during embryonal development, is essential in adult heart tissue, where it is required for long-term preservation of cardiac function [9], [15], [16], so that mutant forms have been associated in a number of studies with the occurrence of cardiomyopathies in man [34]–[36]. For all other tissues, elucidation of critical vinculin involvement still awaits experimental validation by selective knock-out or knock-down approaches. Detection of the splice variant vinculin-ΔEx20, which gives rise to a protein with vinculin-related, unique biochemical properties, opens up the prospect of identifying novel vinculin variants with modified function, which may be associated with pathological conditions.

In conclusion, we here present the detailed characterization of a new variant of the cell adhesion protein vinculin *in vitro* and in mice *in vivo*. The mutant protein lacks sites involved in functionally important interactions with actin as well as with its own head domain turning the mutant insensitive to normal regulatory interactions. The constitutively active behavior of the protein is associated with severe malformations during embryonic development in mice providing *in vivo* evidence for the functional importance of a fine tuned regulation of vinculin function.

## Materials and Methods

### Ethics statement

Mouse breeding and experiments were performed in the animal facilities of the Faculty of Medicine, University of Leipzig according to European (Council Directive 86/609/EEC) and German (Tierschutzgesetz) guidelines for the welfare of experimental animals and were approved by the local authorities (Landesdirection Leipzig). Mice were housed in a 12 h/12 h light dark cycle with access to food and water ad libitum.

### Analysis of mouse embryos

#### Preparation of staged embryos and genotyping

Heterozygous animals were mated and 12–16 hours later females were examined for the presence of a vaginal ejaculatory plug. The day of plug detection was determined as E0.5 of pregnancy. Embryos were isolated at indicated time points. DNA retrieved from the amnions was used for genotyping PCR with primers 5′AGTGAAGACGCCTGTATGG and 5′AGCAGCCCTCTGGAAGGAC. PCR performed for 33 cycles with an annealing temperature of 60°C and 30 s of elongation time resulted in a 271 bp fragment for the wild type and a 324 bp fragment for the transgenic allele comprising the lox-P site.

#### RT-PCR analysis of RNA splicing

Embryos were lysed and RNA was isolated with the RNeasy mini kit (Qiagen) using on-column DNAse digestion. RT reaction was performed with the Revert Aid First strand c-DNA synthesis kit (MBI Fermentas) and the specific primer 5′ GGGAGTCTTTCTGACCCAG 3′ (exon 22). PCR was performed with recombinant Taq-DNA polymerase (MBI Fermentas) using the forward primer 5′ GATGAGCTGGCTCCTCCTAAG 3′ (exon 18), and the reverse primer used for c-DNA synthesis. PCR performed for 39 cycles with an annealing temperature of 60°C and 60 s of elongation time, resulted in 620 bp and 420 bp fragments for wild type and ΔEx20 vinculin mRNA, respectively. The identity of the ΔEx20 mRNA was determined by direct sequencing.

#### Immunoblotting

Embryos were homogenized in lysis buffer (50 mM Tris pH 7.5, 300 mM NaCl, 5 mM EDTA, 1% (w/v) Triton-X-100, 1% (v/v) Protease inhibitor cocktail (Sigma)) and incubated for 30 min on ice. Debris was removed by centrifugation, 13000 rpm at 4°C. Protein concentration in supernatants was determined using the BCA assay (Pierce). Supernatants were diluted with 2x SDS-loading buffer (Sigma) and denatured for 3 min at 95°C. 10 µg total protein extract of each embryo were loaded onto 8% SDS-polyacrylamide gels and separated. After transfer onto nitrocellulose membrane, immunodetection was performed using the vinculin antibody hVin-1 (Sigma) as described below.

#### Embryo embedding and staining

Embryos were fixed using 4% PFA in phosphate buffer over night at 4°C. Thereafter, embryos were washed 3 times in PBS/20 mM glycine, stepwise dehydrated in ethanol and finally embedded in paraffin. 5 µm-sections were taken and mounted onto glass slides. Paraffin sections were stained with haematoxylin and eosin. TUNEL staining was performed according to the manufacturer's protocol (Roche) and counterstained with DAPI (Invitrogen).

#### VCL-ΔIn20/21 (ki/ki) mouse embryonic fibroblasts (MEFs)

To isolate MEF cells from E10.5 embryos, brain and dark red organs were removed. Thereafter, embryos were washed in sterile PBS and cut into smaller pieces. Cells were separated by 0.05% trypsin treatment for 5 min at 37°C. The digest was stopped with FCS and dispersion of cells was enhanced by up and down pipetting through a 1 ml tip. MEFs were seeded onto cell culture dishes and cultured in DMEM (high glucose), 10% FCS, 2 mM L-glutamine and antibiotic/antimycotic mix (GIBCO). Cell preparations from each genotype (passages 2 or 3) were immortalized by retroviral transduction of SV40 LT antigen. Immortalized populations were tested for vinculin expression levels. Their migratory capacity was determined in three-dimensional collagen invasion.

#### Plasmid constructs

Vinculin expression constructs were based on the vinculin full-length clone in pEGFP-C2 (Invitrogen) and the vinculin tail construct (Vt 879–1066) as described earlier [21]. Vinculin exon 20-deletion (ΔEx20) constructs were generated using the QuikChange® site-directed mutagenesis kit (Stratagene), sequenced and subcloned.

### Treatment and analysis of cells

#### Cycloheximide treatment and protein quantification

To assess stability of vinculin transcripts, MEFs were treated with 10 µM cycloheximide, which blocks protein synthesis [37]. Survival of cells was established in a pilot experiment using LDH release as viability marker [38]. For the assay, MEFs were seeded onto 6-well plates (Greiner) and treated the following day with cycloheximide for up to 30 hours. After the treatment, cells were trypsinized, counted and lysed in 2x-SDS sample buffer. Extracts of 60,000 cells were loaded per lane and immunoblotted, using primary antisera for vinculin, hVin-1 (1∶10,000; Sigma), alpha-tubulin (1∶5,000; Sigma), or c-myc (1∶1,000; Millipore) as well as HRP-conjugated secondary serum (1∶10,000; Dianova). Protein extracts for the determination of protein expression in MEFs and transfected C2C12 cells were handled accordingly. GFP-tagged constructs were detected using rabbit anti-GFP (1∶1,000; Invitrogen).

Protein bands were visualized using ECL (GE Healthcare) according to manufacturer's protocol and Amersham Hyperfilm ECL (GE Healthcare). Films were scanned at 600 dpi/16bit greyscale (Epson 2480) and band intensities determined using background subtraction and gel analysis from ImageJ software (National Institutes of Health, http://rsb.info.nih.gov/ij, version 1.42). Graph plots and statistical analysis were carried out using Excel 2004 for Mac (Microsoft).

#### Transfection and immunostaining of MEFs and C2C12 cells

MEFs were seeded onto glass coverslips and transfected (where indicated) on the following day with vinculin expression constructs (0.5 µg DNA) using FugeneHD (Roche) according to manufacturer's protocol. After 24 h of transfection, cells were fixed in phosphate-buffered 4% paraformaldehyd for 10 min at 37°C. Coverslips were washed with PBS/20 mM glycine, permeabilized in PBS/0,01% Tween 20 for 5 min and blocked for 15 min in blocking solution (1% BSA, 5% FCS, 0.05% Triton-X-100). After 1 hour of incubation with the vinculin-specific antibody hVin-1 (Sigma, 1∶800–1,000 in blocking solution), cells were washed in PBS and incubated for 30 min with (i) goat-anti-mouse-alexa-488 conjugated secondary antibody (1∶800–1,000), (ii) DAPI (1∶250) and (iii) alexa-568-conjugated phalloidin (1∶100) in blocking solution (all three Invitrogen). Coverslips were washed in PBS (3×) and water (1×) and mounted using Mowiol (Hoechst). Fluorescence images were taken with a Zeiss Axiovert 200 microscope with HXP 120 illumination using AxioVision 4,7 software (Zeiss).

C2C12 myoblasts [24] were cultured in Dulbecco's modified Eagle's medium (PAA), 10% fetal calf serum (Gibco) and 2 mM L-glutamine (PAA). Subconfluent cells were split and seeded onto fibronectin (30 µg/ml) coated glass coverslips. On the following day, myoblast cells were transfected with vinculin expression constructs using Nanofectin transfection reagent (PAA) according to the manufacturer's instructions and subsequently handled as detailed for MEFs (see above).

#### Adhesion site analysis

Localization and quantification of adhesion parameters, such as "whole cell area", "adhesion number" and "adhesion size" was achieved by processing the fluorescence images of GFP-tagged vinculin variants with ImageJ software. 16bit TIF-images were treated with a cell edge-detection procedure to determine the whole cell area and to limit adhesion quantification to this area. The order of commands for the cell edge-detection was: enhance contrast, FJ edges, make binary, dilate, close, fill holes, and erode. In a second step, the (same) original image was used to select vinculin containing adhesions and to produce the adhesion pattern or footprint. The following commands were used: subtract background, enhance contrast, set threshold, convert to mask. By thresholding, images were reduced to vinculin-containing adhesion patters. "Analyze particles" provided number and size of adhesions from the binary footprint. Graph plots and statistical analysis, normality test and two-tailed unpaired t-test, were carried out using Sigma Plot 10 (Systat).

#### Three-dimensional collagen invasion assay

Preparation of gels and cell invasion analysis were performed as described previously [14]. 100,000 cells were seeded on top of the collagen matrix (2.4 mg/ml) and cultured for 72 hours. At this time period, differences in the invasiveness of cells are clearly visible. After fixation (2.5% glutaraldehyde in PBS), the number of invaded cells and their invasion depth were determined in 12 randomly selected fields of view. To determine the percentage of invaded cells, the adherent cells of the collagen fibre network were also counted. Invasive properties of immortalized MEFs did not change with higher passage number. Statistical analysis of variance was carried out using Sigma Plot 10 (Systat).

#### Fluorescence Recovery After Photobleaching (FRAP)

FRAP experiments were performed as described earlier [24]. In brief, transfected C2C12 cells grown on fibronectin-coated coverslips were observed in an open heating chamber (Warner Instruments, UK) mounted on an Olympus double scan-headed FluoView1000 confocal microscope, equipped with a Plan-Apo 100×/N.A. 1.45 oil objective, and controlled by FV10-ASW software (Olympus). A 488 nm argon laser and 405 nm diode laser were used for EGFP excitation and bleaching, respectively. The two scan heads allowed simultaneous bleaching and image acquisition. The EGFP fluorescence in focal adhesions was bleached 5 frames after movie start (acquisition speed: 1.1 s per frame) by a fast circular movement of the 405 nm laser beam (duration: 500–1000 ms), and recovery of fluorescence recorded over time as indicated. For FRAP analysis, only peripheral focal adhesions of cells expressing medium levels of EGFP-vinculin constructs and sufficient signal-to-noise ratio were selected. FRAP movies were analyzed using ImageJ software. Movies showing clear focus drifts or focal adhesion site growth or dissolution were discarded. Average fluorescence intensities were determined for all frames of the movie in three different regions of interest: the photobleached focal adhesion, background, and the total fluorescent area of the cell. Measured signals were corrected for background fluorescence as well as acquisition photobleaching, and normalized as described [39]. For each recombinant protein, n>10 recovery curves were determined. Statistical analyses and graph plots were carried out using Excel 2004 for Mac (Microsoft) and SigmaPlot 10 (Systat). A bi-exponential regression function describing an exponential rise to a maximum was used for the calculation of half-life times of recovery and protein mobility distributions.

## Supporting Information

Figure S1 Establishment of the VCL-ΔIn20/21 knock-in mouse line. (A) *Genomic structure of the vinculin gene VCL*. The knock-in approach for an inducible expression of vinculin-LD, a lipid binding-deficient mutant of vinculin tail ([26], second half of the targeting construct), required removal of introns 20 and 21 from the targeting vector. A BamH I endonuclease restriction and a lox-P site were inserted into intron 19, 335 bp upstream of exon 20. The targeting construct continued with exons 20, 21 and 22 (no introns) and the 3′UTR. Downstream of the 3′UTR (750 bp), a neomycin resistance cassette flanked by frt-sites, and a second lox-P site were inserted. This sequence was followed by 335 bp of intron 19, a short exon 20–22 cDNA mutated in exons 20 and 22 (star) and another vinculin 3′UTR. The targeting vector was transfected into 129SV embryonic stem cells. (B) *Origin of the mouse line VCL-ΔIn20/21*. Homologous recombination of several ES cell clones was confirmed by Southern Blot analysis. Two positive clones were injected into blastocysts. The VCL-ΔIn20/21 knock-in mouse line was derived from clone 330 and maintained by intercrossing of heterozygous animals. A deleter mouse harbouring FLPe [40] was employed to remove the Neo cassette. 40. Rodriguez CI, Buchholz F, Galloway J, Sequerra R, Kasper J, et al. (2000) High-efficiency deleter mice show that FLPe is an alternative to Cre-loxP. Nat Genet 25: 139–140.
(0.60 MB TIF)

Figure S2 Alternative splicing of the VCL-ΔIn20/21 allele. (A) *Sequence analysis of the alternative splice site*. RT-PCR using primers in exons 18 and 22 was employed to obtain a cDNA fragment of vinculin-ΔEx20 from E10.5 total RNA. DNA sequencing revealed mRNA and derived amino acid sequences of the exon 18 to 21 boundary. (B) *Pre mRNA of the VCL-ΔIn20/21 allele*. Exon 20 contains consensus nucleotide sequences required for conventional intron splicing [41]. The proposed branch site ‘CUPuAPy’ (green) and the splice acceptor site ‘CAG/G’ (underlined) including pyrimidine-rich region (blue) are indicated. The splice donor site of exon 18 is maintained (not shown). (Py: C or U; Pu: A or G). 41. Alberts B, Johnson A, Lewis J, Raff M, Roberts K, Walter P (2002) Molecular biology of the cell (4th edition), Garland Science, New York. 319–324p.
(0.31 MB TIF)

Figure S3 Protein stability of wild type and ΔEx20 vinculin in MEFs. (A) *Representative immunoblots of vinculin and c-myc*. VCL-ΔIn20/21 MEFs were treated with 10 µM cycloheximide (CHX) for the indicated periods of time [37]. Extracts of 60,000 cells (each) were loaded and immunoblotted. Tubulin signals served as loading/transfer controls. Note loss of c-myc after 6 hours of treatment. Wild type vinculin (solid line) remained stable over 30 hours in all MEF genotypes (only (ki/ki) MEFs are shown), whereas vinculin-ΔEx20 (broken line) was reduced to 54% (n = 4). (B) Exponential fit of protein levels (broken line; y = 0.92 * e ^−0.02 x^ , R^2^ = 0.90) provides an estimate of the vinculin-ΔEx20 half life time of 1.5 days (35 hours) in cells. Error bars: S.D.
(1.05 MB TIF)
