# Supplementary figures and images for: The Vinculin-ΔIn20/21 Mouse: Characteristics of a Constitutive, Actin-Binding Deficient Splice Variant of Vinculin
Source: PLoS One. 2010 Jul 14;5(7):e11530. doi: 10.1371/journal.pone.0011530 (PMC2904371; doi:10.1371/journal.pone.0011530)

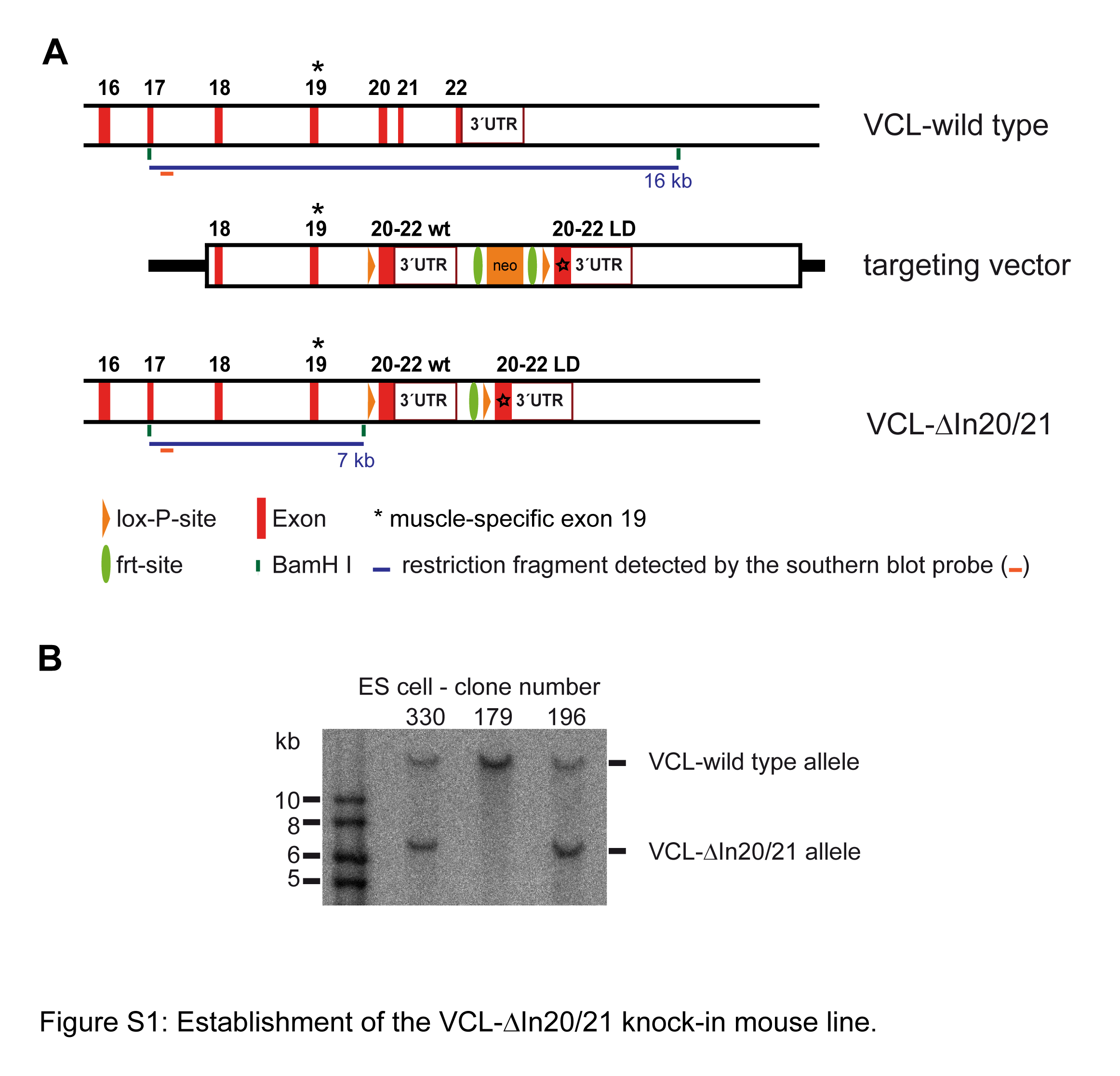

Supplement: Figure S1 — Establishment of the VCL-ΔIn20/21 knock-in mouse line. (A) Genomic structure of the vinculin gene VCL. The knock-in approach for an inducible expression of vinculin-LD, a lipid binding-deficient mutant of vinculin tail ([26], second half of the targeting construct), required removal of introns 20 and 21 from the targeting vector. A BamH I endonuclease restriction and a lox-P site were inserted into intron 19, 335 bp upstream of exon 20. The targeting construct continued with exons 20, 21 and 22 (no introns) and the 3′UTR. Downstream of the 3′UTR (750 bp), a neomycin resistance cassette flanked by frt-sites, and a second lox-P site were inserted. This sequence was followed by 335 bp of intron 19, a short exon 20–22 cDNA mutated in exons 20 and 22 (star) and another vinculin 3′UTR. The targeting vector was transfected into 129SV embryonic stem cells. (B) Origin of the mouse line VCL-ΔIn20/21. Homologous recombination of several ES cell clones was confirmed by Southern Blot analysis. Two positive clones were injected into blastocysts. The VCL-ΔIn20/21 knock-in mouse line was derived from clone 330 and maintained by intercrossing of heterozygous animals. A deleter mouse harbouring FLPe [40] was employed to remove the Neo cassette. 40. Rodriguez CI, Buchholz F, Galloway J, Sequerra R, Kasper J, et al. (2000) High-efficiency deleter mice show that FLPe is an alternative to Cre-loxP. Nat Genet 25: 139–140. (0.60 MB TIF) [file pone.0011530.s001.tif]

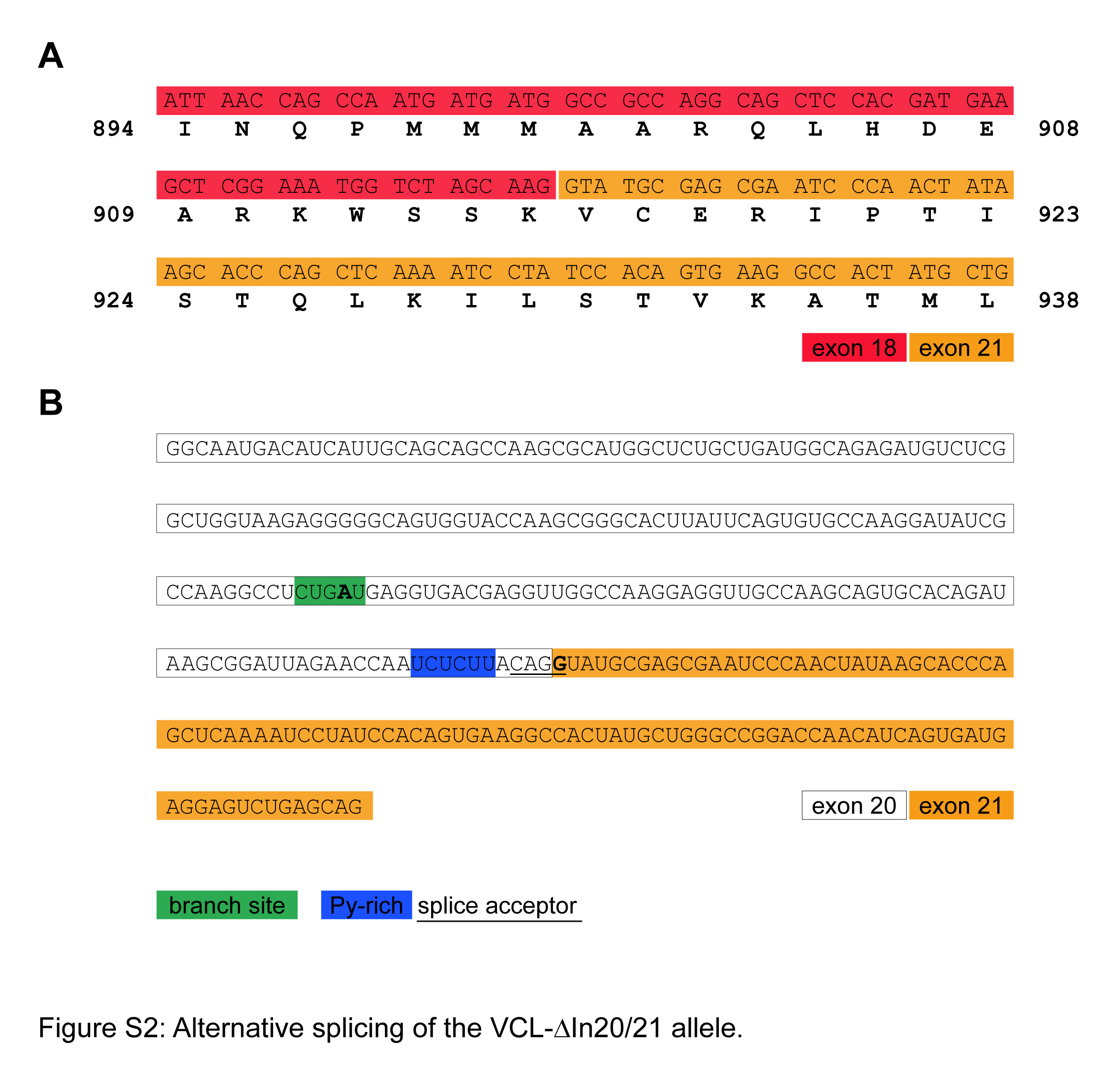

Supplement: Figure S2 — Alternative splicing of the VCL-ΔIn20/21 allele. (A) Sequence analysis of the alternative splice site. RT-PCR using primers in exons 18 and 22 was employed to obtain a cDNA fragment of vinculin-ΔEx20 from E10.5 total RNA. DNA sequencing revealed mRNA and derived amino acid sequences of the exon 18 to 21 boundary. (B) Pre mRNA of the VCL-ΔIn20/21 allele. Exon 20 contains consensus nucleotide sequences required for conventional intron splicing [41]. The proposed branch site ‘CUPuAPy’ (green) and the splice acceptor site ‘CAG/G’ (underlined) including pyrimidine-rich region (blue) are indicated. The splice donor site of exon 18 is maintained (not shown). (Py: C or U; Pu: A or G). 41. Alberts B, Johnson A, Lewis J, Raff M, Roberts K, Walter P (2002) Molecular biology of the cell (4th edition), Garland Science, New York. 319–324p. (0.31 MB TIF) [file pone.0011530.s002.tif]

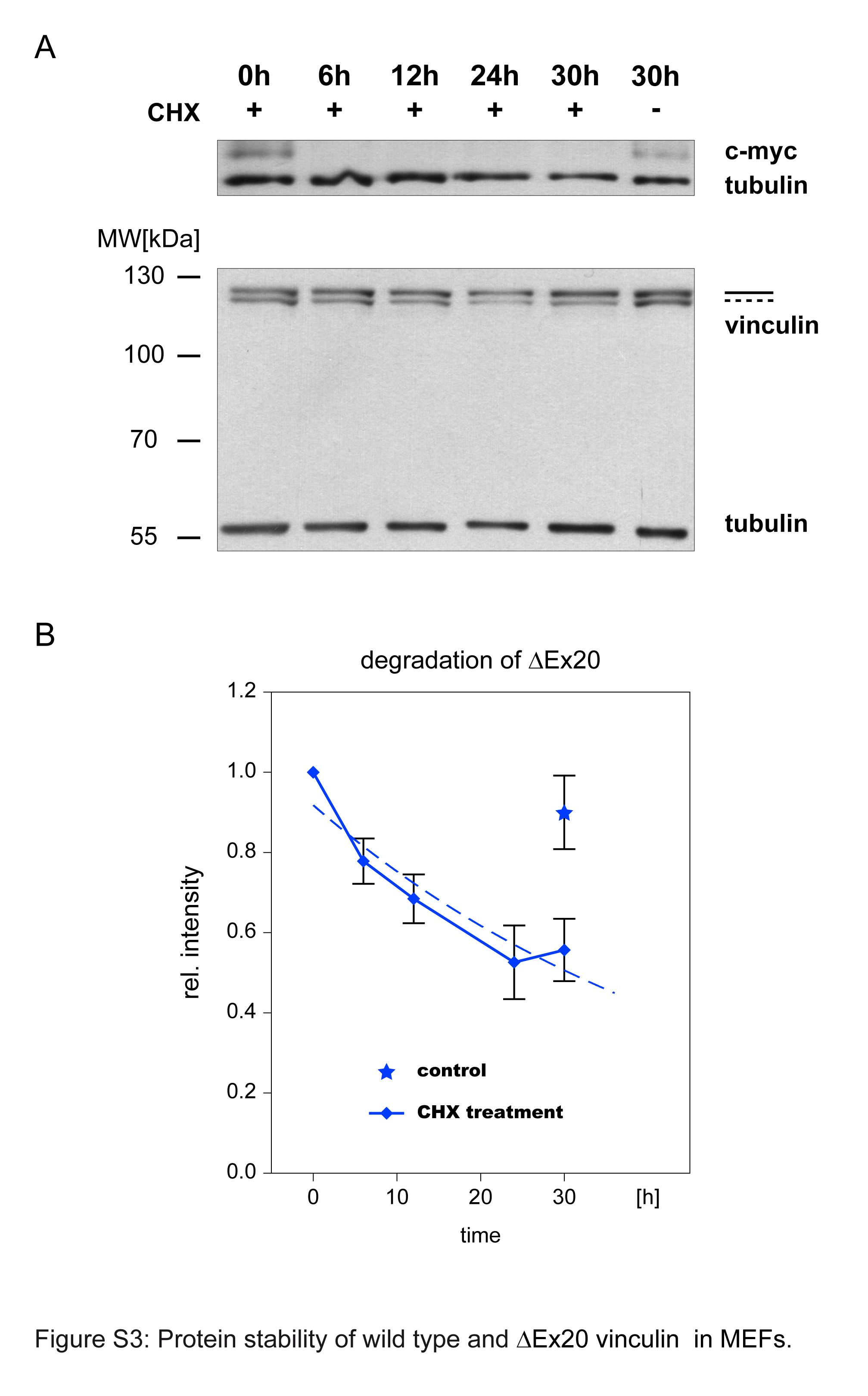

Supplement: Figure S3 — Protein stability of wild type and ΔEx20 vinculin in MEFs. (A) Representative immunoblots of vinculin and c-myc. VCL-ΔIn20/21 MEFs were treated with 10 µM cycloheximide (CHX) for the indicated periods of time [37]. Extracts of 60,000 cells (each) were loaded and immunoblotted. Tubulin signals served as loading/transfer controls. Note loss of c-myc after 6 hours of treatment. Wild type vinculin (solid line) remained stable over 30 hours in all MEF genotypes (only (ki/ki) MEFs are shown), whereas vinculin-ΔEx20 (broken line) was reduced to 54% (n = 4). (B) Exponential fit of protein levels (broken line; y = 0.92 * e −0.02 x , R2 = 0.90) provides an estimate of the vinculin-ΔEx20 half life time of 1.5 days (35 hours) in cells. Error bars: S.D. (1.05 MB TIF) [file pone.0011530.s003.tif]
